# Volitional control of walking in aging

**DOI:** 10.18632/aging.203986

**Published:** 2022-03-28

**Authors:** Roee Holtzer

**Affiliations:** 1 Ferkauf Graduate School of Psychology, Yeshiva University, Bronx, NY 10461, USA; 2 Department of Neurology, Albert Einstein College of Medicine, Bronx, NY 10461, USA

**Keywords:** gait, brain, functional near-infrared spectroscopy (fNIRS), magnetic resonance imaging (MRI), neural inefficiency

Walking ability is a robust predictor of health outcomes in aging. Compelling evidence supports meaningful interrelations between walking and cognition, notably when the former is performed under dual-task conditions that place increased demands on attention and executive control resources subserved by the prefrontal cortex (PFC) [1]. Indeed, a recent review and meta-analytic study established that due to the increased cognitive demands involved in allocating attention to competing task demands, older adults demonstrate a reliable decline in walking performance in dual- compared to single-task conditions [2]. Importantly, dual-task walking paradigms approximate conditions in natural environments that require adaptation to competing sensory, physical and cognitive interferences.

Due to the limitations inherent in traditional neuroimaging methods such as magnetic resonance imaging (MRI), our ability to understand and quantify how the brain is functionally involved in gait control during active walking has been limited. Nonetheless, recent studies using functional Near-Infrared Spectroscopy (fNIRS), a noninvasive optics-based neuroimaging modality, have begun to shed light on the functional brain correlates of walking. This technology uses light in the near infrared range (650-950nm) to quantify changes in oxygenated (HbO_2_) and deoxygenated hemoglobin (Hb) and is uniquely suited to examine task-related changes in brain hemodynamic responses during active walking [3]. A recent review and meta-analytic study demonstrated a reliable increase in fNIRS-derived activation in the PFC in dual- compared to single-task walking conditions in aging and clinical populations confirming the key role of this brain region in cognitive control of locomotion under attention-demanding conditions [4].

While the involvement of the PFC in cortical control of walking, notably under cognitively demanding conditions is well-established, it is important to recognize that limited spatial resolution and depth of penetration of fNIRS technology constrain the scope of investigations designed to interrogate brain systems that support locomotion. To address these limitations, we have neuroimaged participants using independent MRI and fNIRS protocols to determine how structural and functional brain systems interact vis-à-vis walking. The neural inefficiency hypotheses served as a conceptual framework to examining how structural brain integrity might influence hemodynamic changes in the PFC across walking task conditions. Neural inefficiency exists in persons exhibiting higher brain activation but equivalent or worse performance than their counterparts and can be optimally examined in dual-task walking where cognitive demands are experimentally increased compared to single-task walking. We have previously shown that neural efficiency under dual-task walking conditions was improved with practice (i.e., fNIRS-derived activations in the PFC were reduced and walking performance improved after repeated trials) [5]. Our studies utilizing independent MRI and fNIRS protocols revealed that, among older adults, worse white matter integrity assessed using whole brain fractional anisotropy [6] and smaller gray matter volume, notably but not exclusively in the frontal cortex [7], were associated with inefficient increases in fNIRS-derived HbO_2_ in dual- compared to single-task walking conditions. These findings suggested that poor structural integrity of the brain compromised the efficiency of the neural response to increased cognitive demands of walking.

Cortical thickness and gray matter volume are genetically and phenotypically distinct. Therefore, our recent investigation was designed to determine the effect of cortical thickness on changes in the hemodynamic response in the PFC across walking task conditions to further elucidate interactions between structural and functional brain systems of locomotion [8]. Community-residing older adults enrolled in a longitudinal study designed to determine cognitive and brain predictors of mobility were eligible to participate in this investigation. The parent study cohort, inclusion/exclusion criteria, and procedures were described in numerous publications. Briefly, community-residing and ambulatory older adults age ≥65 years were eligible to participate. Inability to speak English, dementia, significant impairments in vision or hearing, history of a neurological or psychiatric disorders, current medical procedures that would hinder ambulation, and current hemodialysis treatment served as exclusion criteria. Cognitive status (normal, mild cognitive impairments, dementia) was determined via established consensus clinical case conference procedures. The subsample (n=55; mean age in years=74.84 ± 4.97; %females =49.1%) was comprised of dementia-free participants who underwent the combined fNIRS dual-task walking paradigm and separately completed the MRI protocol. Details concerning the fNIRS dual-task walking protocol have been provided in previous publications. Briefly, in the Single-Task-Walk condition, participants were asked to walk around a 4 x 20 foot Zeno electronic walkway for three continuous counterclockwise loops at their “normal pace”. In the Dual-Task-Walk condition participants were instructed to walk while reciting alternate letters of the alphabet at the same time. Changes in HbO_2_ levels in the PFC across experimental conditions were assessed during active walking using fNIRS Imager 1100 (fNIR Devices, LLC, Potomac, MD). Magnetic resonance imaging was performed in a 3 T Phillips scanner (Achieva TX; Philips Medical Systems, Best, The Netherlands). The scanner was equipped with a 32-channel head coil. FreeSurfer software package (http://surfer.nmr.mgh.harvard.edu/) was used to extract cortical thickness measures in 68 brain regions (34 in each hemisphere). The results revealed that thinner cortex in specific regions in the frontal, temporal, parietal and occipital lobes as well as the cingulate cortex and insula was associated with inefficient increases in fNIRS-derived HbO_2_ from single to dual-task walking conditions, notably the analyses controlled for several covariates including gait and cognitive performance. These findings indicate that multiple brain regions are involved in volitional control of walking in older adults. Furthermore, reduced cortical thickness maybe an important causal factor implicated in neural inefficiency in aging and possibly in other clinical populations.
